# Knee Dislocation as a Result of a Ski Injury

**DOI:** 10.7759/cureus.62841

**Published:** 2024-06-21

**Authors:** Hoomaan Ebadi, Landan Banks, Morteza Khodaee

**Affiliations:** 1 Orthopaedics, School of Medicine, Shahid Beheshti University of Medical Sciences, Tehran, IRN; 2 Family Medicine, University of Colorado School of Medicine, Aurora, USA

**Keywords:** reduction, sport, multiple ligaments knee injury, trauma, knee deformity

## Abstract

Visible knee deformity as a result of a sporting activity is rare; however, it can be caused by a serious injury and have catastrophic consequences. Differential diagnosis includes patellofemoral or knee dislocations, fractures, and tendon or ligament ruptures. Immediate diagnosis and appropriate management are key. Diagnosis can be made using available tools such as plain radiography, ultrasound, CT scan, and MRI. Depending on the type and severity of the diagnosis, urgent transportation to a higher level of care facility may be indicated. We present a gentleman in his 20s with knee dislocation as a result of a ski injury. His knee was reduced and he was transported to a hospital with surgical capability. He underwent surgery to stabilize his injury and then staged reconstruction for rupture of multiple ligaments.

## Introduction

Visible knee deformity is a rare condition in sports [1-4]. Knee deformity can be a result of a direct (e.g. collision with an external object) or indirect (e.g. twisting) trauma. Fractures and dislocations around the knee, hematoma, hemarthrosis, and major ligament ruptures can cause knee deformity. As the differential diagnosis of a visible knee deformity includes some limb-threatening conditions, a timely and appropriate management strategy is critical. Reviewing the mechanism of injury and performing a comprehensive and focused physical examination is the key to narrow the differential diagnosis [1-4]. Inability to bear weight or ambulate is an alarming sign [2,3]. Frequent neurovascular evaluation should be performed, particularly if more serious injuries such as knee dislocation or fractures are suspected [2,3,5-8]. In case of deteriorating neurovascular status, an urgent intervention may be necessary. We present and discuss the evaluation and management of a patient with a visible knee deformity as a result of a ski injury.

## Case presentation

**Figure 1:**
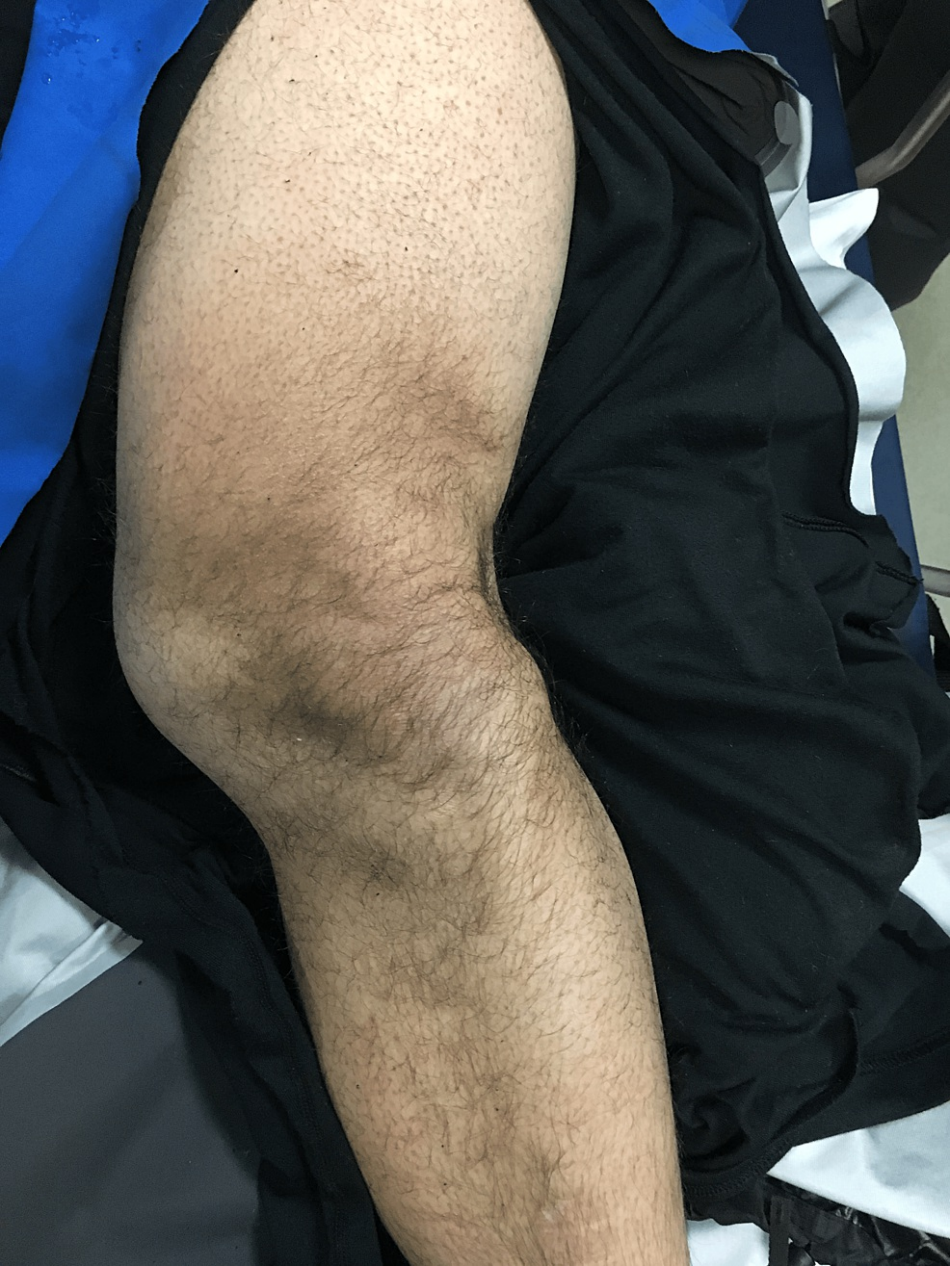
Left lower leg deformity as a result of a ski injury.

A gentleman in his 20s was brought to the clinic at the bottom of a ski resort. About an hour earlier, he hit a rock while skiing and fell forward and his ski became lodged between two trees. He felt immediate pain in his left knee. He was unable to bear weight and was transported to the clinic by ski patrols. He was helmeted during the incident and denied head trauma or loss of consciousness. He acquired no other injuries. He denied numbness or weakness in the left lower extremity. He had no previous injuries to that leg. His vital signs were normal. On physical examination, there was a deformity of the left knee (Figure 1).

**Figure 2:**
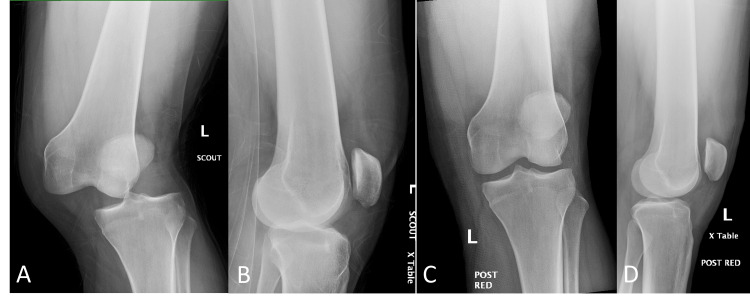
Lateral displacement of the left tibia and fibula in relation to the femur in anteroposterior (A) and lateral (B) radiographic views. Post-reduction images reveal anatomical alignment (C, D).

The left foot was dusky with poor capillary refill of 2 to 3 seconds but had brisk pulses. Dorsalis pedis and posterior tibial pulses were 2+ bilaterally with a normal ankle-brachial index (ABI). There was no sensory deficit. Sensation and ability to move the ankle and toes were within normal limits. Plain radiography showed displacement of the tibia and fibula in relation to the femur (Figures 2A, 2B). His knee was reduced (Figures 2C, 2D) with conscious sedation using intravenous propofol.

**Figure 3:**
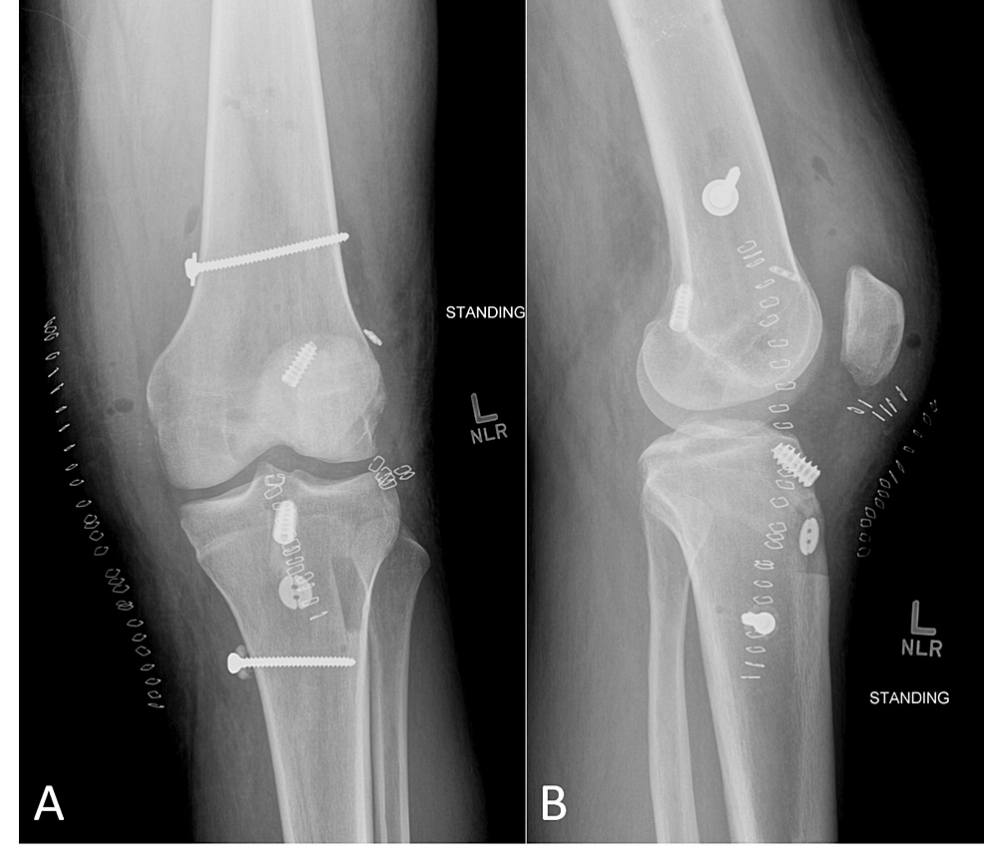
Post-operative radiographic appearance of left knee following anterior cruciate ligament, medial collateral ligament, and posterior cruciate ligament reconstruction (A, B). The instrumentation appears well seated without evidence of convocation.

His lower extremity neurovascular examinations remained normal. His knee was placed in a splint immobilizer and he was transferred via ambulance to a higher level of care facility 90 minutes away. CT angiogram found no vascular injury. MRI showed extensive ligamentous disruption including anterior cruciate ligament (ACL), posterior cruciate ligament (PCL), and medial collateral ligament (MCL) ruptures. An external fixation system was placed in order to keep his knee in a stable, reduced position until further management. Due to gross instability of the knee, it was decided to perform a staged reconstruction. A four-week course of pre-operative rehabilitation (prehabilitation) was completed before definitive reconstruction of the ACL, PCL, and MCL (Figure 3).

## Discussion

Dislocation of the knee is a rare, but serious injury which may result in long-term physical and psychological impairment [1-3,9]. This occurs when the distal femur and proximal tibia lose articulation with each other. Usually, there are multiple ligamentous ruptures [1,5,9]. About half of knee dislocations are the result of road traffic accidents and a third are due to sporting events (e.g. high velocity and contact sports) [2,5,9]. The most common types are anterior and posterior dislocations accounting for up to 70% of the cases [1,5,9,10]. Medial and lateral knee dislocations are less common [1,5,9,10]. Up to half of the knee dislocations are spontaneously reduced making the diagnosis challenging [1,5,9,10]. Therefore, any multiligament knee injuries should be assumed as a spontaneously reduced knee dislocation [1,9,10]. About 20% of patients with knee dislocations may develop neurovascular injuries [1,5,9-14] Injuries to the popliteal artery and common peroneal nerve are the most commonly reported complications [9,11,12].

Differential diagnoses of patients with traumatic knee deformity

Differential diagnoses of patients with traumatic knee deformity include fractures, dislocation, and ligament/tendon ruptures. Other potential etiologies include soft tissue injuries such as hemorrhagic prepatellar bursitis, Morel Lavallee lesions, and massive hemarthrosis [2,7,8].

Patellar (Patellofemoral) Dislocation

This is a relatively common condition that occurs when the patella dislocates (typically laterally) from its normal position in the trochlear groove of the femur [2]. On physical examination, the anterior part of the knee shows a deformity and the patient usually is unable to flex or extend the knee [2,3]. Plain radiography, particularly a sunrise view, is diagnostic. Reduction is achieved with gentle knee extension and pressure to the lateral aspect of the patella [2,3].

Fractures Around the Knee

Fractures of distal femur, patella, tibia, and fibula can cause major deformity [2]. Patients with any suspicions of fractures should be evaluated and at least plain radiography should be obtained [2,3].

Patellar and Quadriceps Tendon Ruptures

These injuries usually occur as a result of a non-contact eccentric load (e.g. landing after a high jump) or kicking [2,3]. On physical examination, there is usually a significant hemarthrosis and inability to extend the knee [2,3]. There is usually a palpable gap below the patella (patellar tendon rupture) or in the suprapatellar area (quadriceps tendon rupture) [2,3]. Plain radiography may reveal patella alta in cases with patellar tendon rupture [2,3]. The patient’s knee should be immobilized and a referral to orthopedic surgery should be arranged [2,3].

Isolated ACL Rupture

This injury may occur as a result of a contact or non-contact injury [2,3]. There is usually no obvious deformity other than significant knee joint effusion (hemarthrosis) [2,3]. The patient is usually able to flex and extend the knee and often has an unstable gait [3]. 

Evaluation

Evaluation of a patient with suspected knee dislocation should follow the Advanced Trauma Life Support (ATLS) primary and secondary survey protocols [1,5,9,10]. Physical examination should include palpation of the distal pulses, ABI, and neurologic sensory and motor testing [1,5,9,10]. In patients with an unstable knee and with suspected multiligament ruptures, there should be a high index of suspicion for a spontaneously reduced knee dislocation [1,5,9,10]. If there is a deformity, plain radiography images should be obtained to evaluate for any fractures or dislocations [1,2,5,9,10].

Management

Reduction should be attempted if knee dislocation is confirmed by plain radiography [1,5,9]. This should be done under adequate analgesia and sedation. Lateral and medial dislocations are generally difficult to reduce [1,5,9]. Transportation of the patient to a facility with advanced care should not be delayed in order to achieve reduction [1,2,5,9]. Serial post-reduction neurovascular examinations should be performed [9-11,13]. The knee should be immobilized and the patient should be immediately transferred to a higher level of care facility with access to surgical intervention. Any suspected vascular injury should be investigated with CT or MR angiography [1,5,9-11,13]. Consultations with vascular and orthopedic surgery teams should be pursued.

## Conclusions

Visible knee deformity as a result of a trauma is rare, but if it is not managed appropriately, it can have catastrophic consequences. A proper diagnosis using available resources such as radiography or advanced imaging is critical. If the diagnosis is consistent with a possible severe injury such as knee dislocation or complex fracture around the knee, management should include timely transportation to a facility with higher level of care. Frequent neurovascular evaluation is critical and patients may need an urgent operation performed by a surgical group consisting of an orthopedic trauma surgeon and a vascular surgeon. Prognosis after these severe injuries, if treated appropriately, is generally good. 
